# Interplay between ADP-ribosyltransferases and essential cell signaling pathways controls cellular responses

**DOI:** 10.1038/s41421-021-00323-9

**Published:** 2021-11-02

**Authors:** Flurina Boehi, Patrick Manetsch, Michael O. Hottiger

**Affiliations:** 1 Department of Molecular Mechanisms of Disease, University of Zurich, Zurich, Switzerland; 2 Cancer Biology PhD Program of the Life Science Zurich Graduate School, University of Zurich, Zurich, Switzerland; 3 Molecular Life Science PhD Program of the Life Science Zurich Graduate School, University of Zurich, Zurich, Switzerland

**Keywords:** Cell signalling, Post-translational modifications

## Abstract

Signaling cascades provide integrative and interactive frameworks that allow the cell to respond to signals from its environment and/or from within the cell itself. The dynamic regulation of mammalian cell signaling pathways is often modulated by cascades of protein post-translational modifications (PTMs). ADP-ribosylation is a PTM that is catalyzed by ADP-ribosyltransferases and manifests as mono- (MARylation) or poly- (PARylation) ADP-ribosylation depending on the addition of one or multiple ADP-ribose units to protein substrates. ADP-ribosylation has recently emerged as an important cell regulator that impacts a plethora of cellular processes, including many intracellular signaling events. Here, we provide an overview of the interplay between the intracellular diphtheria toxin-like ADP-ribosyltransferase (ARTD) family members and five selected signaling pathways (including NF-κB, JAK/STAT, Wnt-β-catenin, MAPK, PI3K/AKT), which are frequently described to control or to be controlled by ADP-ribosyltransferases and how these interactions impact the cellular responses.

## Introduction

Communication via signals is a dynamic ability innate to every living organism. It enables cells to receive and process signals, not only from the external environment but also from discrete regions (i.e., organelles) within the cell^1^. In multicellular organisms, communication between cells is mainly mediated by extracellular signaling molecules. Binding of the signal molecule, or ligand, to the corresponding receptor activates one or more intracellular signaling pathways^2^. These pathways often comprise a cascade of specific proteins that transduce or amplify the incoming signal, ultimately regulating the activity of effector proteins which modulate various cellular functions and behaviors^3^. Enzymes play a critical role in this process since they can transmit the upstream signal by generating second messengers or modifying and activating a downstream protein component of the pathway. Many of these enzymes behave like molecular switches whose activation state is regulated by post-translational modifications (PTMs)^4^.

In recent years, ADP-ribosylation has emerged as a complex, dynamic, and reversible PTM that impacts the regulation and maintenance of many cellular processes, including intracellular signaling events^5–7^. ADP-ribosyltransferases (ARTs) catalyze the transfer of ADP-ribose moieties from nicotinamide adenine dinucleotide (NAD^+^) to a diverse range of target molecules including proteins, nucleic acids and small molecules^8–11^. While the transfer of only one ADP-ribose unit is called mono-ADP-ribosylation (i.e., MARylation), the already bound unit can be elongated by the incorporation of additional ADP-ribose units, called poly-ADP-ribosylation (i.e., PARylation)^12–14^. Mammalian ARTs have classically been divided into three families: (i) clostridium toxin-like ARTs (ARTCs) are mainly described to catalyze extracellular ADP-ribosylation, while (ii) diphtheria toxin-like ARTs (ARTDs) and (iii) selected members of the Sirtuins family (i.e., Sirt 4, 6, and 7) catalyze ADP-ribosylation in different intracellular compartments^14–16^. Here, we will focus on intracellular ARTD family members, that comprises 17 enzymes in humans^15,17^. According to a recently agreed consensus, we use “PARP” as a name on its own to describe the different ARTD family members.

NAD^+^, the only known co-substrate for ADP-ribosylation, as well as its reduced form NADH, are essential molecules involved in regulating a plethora of cellular processes including cellular energy metabolism, gene expression, inflammation, aging, carcinogenesis, and cell death, thus linking ADP-ribosylation to these processes^6,7,18^. Moreover, NAD^+^ availability is tightly linked to the subcellular distribution of NAD^+^ pools. Due to the fact that NAD^+^ is cell membrane impermeable, NAD^+^ is compartmentalized in separate pools/organelles that differ in their NAD^+^ concentration and it is regulated by NAD^+^ transporters^18–22^. The concentration of free NAD^+^ in different subcellular compartments ranges from 87 to 136 μM for the nucleus and cytoplasm to 191–300 μM in the mitochondria^7,23–25^. While the majority of intracellular NAD^+^/NADH is protein bound, the unbound proportion of NAD^+^ exceeds free NADH by ~600–1000 times in the cytosol and 7–8 times in the mitochondria^7,18,19^. The concentration of cellular NAD^+^ not only depends on the consuming enzymes, but also on the rate of its synthesis. NAD^+^ can be synthesized de novo from tryptophan, from nicotinic acid (NA) in the Preiss-Handler pathway or from nicotinamide (NAM) in the salvage pathway^17,25^. Since the ARTD family members belong to the major consumers of free NAD^+^ in the cell this, ultimately, implies that the localization of ARTD family members, their *K*_m_ NAD^+^ and expression levels as well as potential activators (e.g., DNA damage or co-factors) influence the ADP-ribosylation levels^17^. Most ARTD family members localize either to the nucleus or the cytoplasm, except for PARP12 (ARTD12) and PARP16 (ARTD15), which localize to the endomembrane system (Table 1). While the *K*_m_ NAD^+^ of most ARTDs is within the range of the nuclear and cytoplasmic NAD^+^ concentration, the free NAD^+^ concentration is especially important for ARTD family members with a *K*_m_ NAD^+^ higher than the NAD^+^ concentration in the corresponding compartment (Table 1)^17,26^. As a result, the activity of those ARTD family members relies either on a local increase of the NAD^+^ concentration^17^ or the absence of competing ARTs with a lower *K*_m_ NAD^+^ ^27,28^. This suggests that NAD^+^ availability might under certain circumstances be a limiting factor that controls the catalytic capacity of ARTs and subsequently the regulation of signaling pathways by ARTD-mediated ADP-ribosylation.

**Table 1:** Human ARTD family members and their involvement in selected intracellular signaling pathways, their cellular localization and catalyzed products as well as their affinity for NAD^+^ ^7,17,26,28^.

ARTD family members	Alternative name	Signaling pathways	Cellular localization	Catalytic activity	*K*_M_ NAD^+^ (μM)
PARP1	ARTD1	NF-kB, JAK/STAT, Wnt-β-catenin, MAPK, PI3K/AKT	Nucleus	PARylation	35 ± 15^a^
PARP2	ARTD2	PI3K/AKT	Nucleus	PARylation	159 ± 2
PARP3	ARTD3	PI3K/AKT	Nucleus	MARylation	131 ± 57
PARP4	ARTD4	–	Cytoplasm	MARylation (PARylation?)	92 ± 17
Tankyrase 1	ARTD5, PARP5a	Wnt-β-catenin, MAPK, PI3K/AKT	Cytoplasm	PARylation	31 ± 4
Tankyrase 2	ARTD6, PARP5b	Wnt-β-catenin	Cytoplasm	PARylation	251 ± 56
PARP6	ARTD17	–	Cytoplasm, Nucleus	MARylation	N.D.
PARP7	ARTD14, TIPARP	NF-kB	Nucleus	MARylation	N.D.
PARP8	ARTD16	–	Cytoplasm, Nucleus	MARylation	N.D.
PARP9	ARTD9, BAL1	JAK/STAT	Cytoplasm, Nucleus	MARylation	197 ± 64
PARP10	ARTD10	NF-kB, Wnt-β-catenin	Cytoplasm, Nucleus	MARylation	98 ± 11
PARP11	ARTD11	NF-kB, JAK/STAT	Nuclear envelope	MARylation	N.D.
PARP12	ARTD12	NF-kB	Golgi	MARylation	299 ± 76
PARP13	ARTD13	NF-kB	Cytoplasm	inactive	inactive
PARP14	ARTD8, BAL2	NF-kB, JAK/STAT, PI3K/AKT	Cytoplasm, Nucleus	MARylation	62 ± 7
PARP15	ARTD7	–	Nucleus	MARylation	11 ± 4.2
PARP16	ARTD15	NF-kB	Endoplasmic reticulum	MARylation	582 ± 196

^a^The *K*_M_ NAD^+^ value of PARP1 is regulated by co-factors and the presence of DNA.

Here we will summarize and discuss the interplay between different ARTDs and five selected signaling pathways that have been most frequently described to control or be controlled by ARTDs: NF-κB, JAK/STAT, Wnt-β-catenin, MAPK, and PI3K/AKT signaling cascades. In our description we will follow the signaling cascades starting at the plasma membrane and emphasize the importance or negative impact of the so far describe ARTD family members for the respective pathway components. Moreover, we included the importance of the regulatory function of the cellular NAD^+^ levels when appropriate in the chapter or in the concluding paragraph.

## Interplay between NF-κB signaling and ARTD family members

### Overview of the NF-κB pathway

**Fig. 1:**
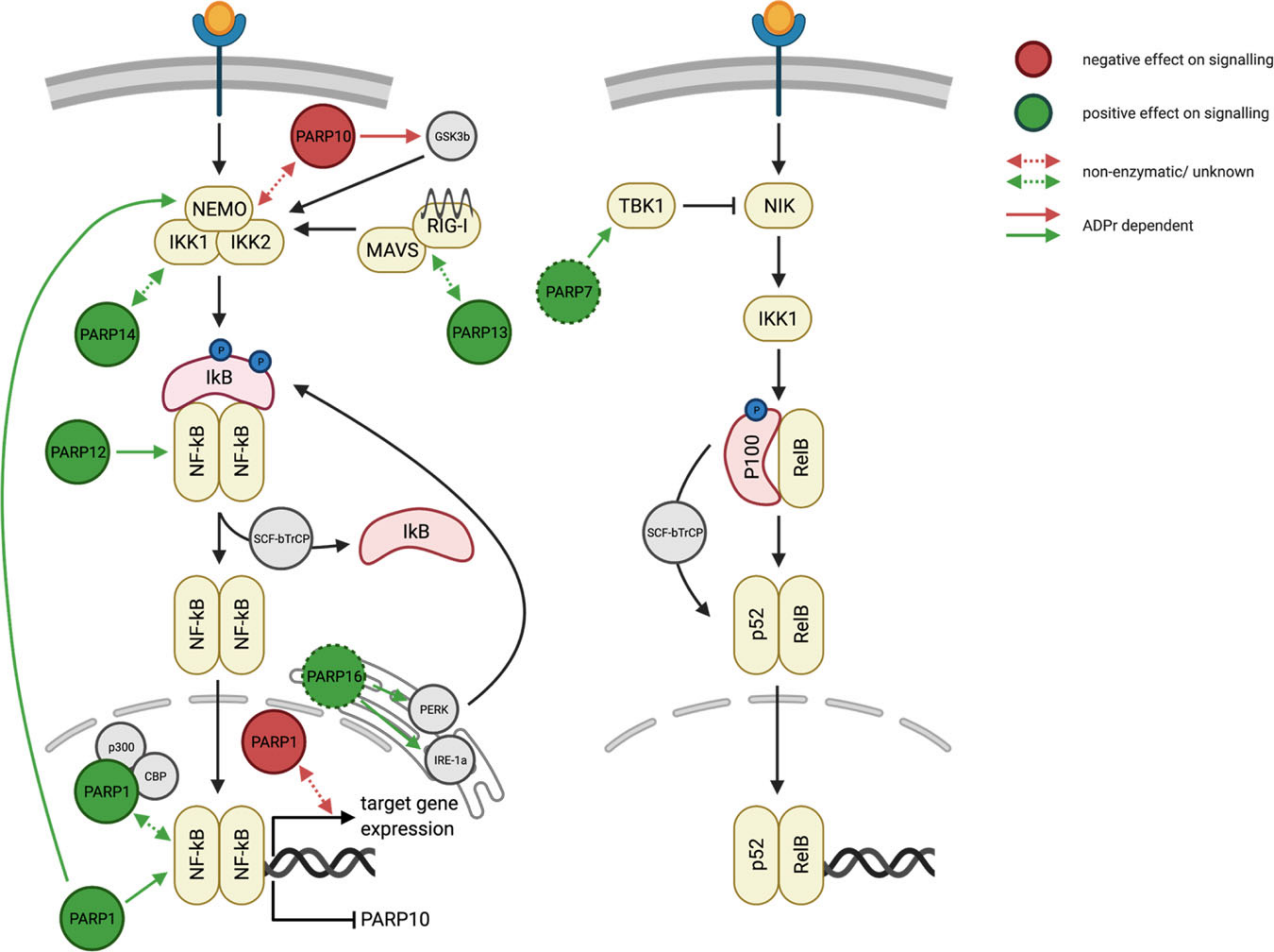
Schematic overview of the interplay between NF-κB signaling and ARTD family members. Canonical (left panel) and non-canoncial (right panel) pathway. Positive regulations of NF-κB signaling by ARTD family members are depicted in green, while negative effects of ARTDs on the signaling pathway are shown in red. Solid lines indicate the contribution of ADP-ribosylation to the regulation of NF-κB signaling. In case the contribution of ADP-ribosylation was not described or the protein itself rather than its enzymatic activity is involved in the regulation, the interactions are represented by dashed lines.

The cellular nuclear factor-κB (NF-κB) signaling pathway is a central element of multiple physiological and pathological processes^29^ and is induced by two major pathways, the canonical and the non-canonical one (Fig. 1)^30^.

The canonical NF-κB pathway is activated in response to diverse stimuli, including ligands of various cytokine receptors, pathogen recognition receptor (PRRs), TNF receptor (TNFR) superfamily members, as well as T-cell receptor (TCR) and B-cell receptor^30^. In the inactive state, IκBα sequesters and retains NF-κB in the cytoplasm. NF-κB is composed of five structurally related members, including NF-κB1 (p50), NF-κB2 (p52), RelA (p65), RelB, and c-Rel^29^. These subunits form diverse homo- and heterodimers which convey different transcriptional outputs upon activation. Signaling through the canonical pathway, initiated by ligand binding, results in the site-specific phosphorylation and proteasomal degradation of IκBα induced by the multi-subunit IκB-kinase (IKK) complex, resulting in the release of NF-κB^31,32^. The IKK complex is composed of two kinase subunits, IKK1 (IKKα) and IKK2 (IKKβ), and a structural subunit NEMO (IKKγ)^31^. The release of NF-κB is followed by nuclear translocation of NF-κB dimers. Resulting in a rapid and transient transcriptional activation of target genes^32^.

In contrast, the non-canonical pathway integrates signals from a subset of TNF receptor family members and results in the activation of NF-κB-inducing kinase (NIK)^33^. NIK activates IKK1, which induces processing of p100, a RelB-specific inhibitor and the precursor of NF-κB2 (p52). Ultimately, this results in the nuclear translocation of NF-κB2-RelB dimers and induces a slow but persistent transcriptional re-programming^33^.

### Influence of NF-κB signaling on ARTD family member expression

NF-κB was described to bind to and inhibit the *PARP10* (*ARTD10*) promoter leading to its transcriptional repression in hepatocellular carcinoma^34^. In accordance with these results, inhibition of the IKK complex, which inactivates NF-κB signaling, strongly increased PARP10 mRNA and protein levels. The p65-dependent repression of the *PARP10* promoter was confirmed by the overexpression of p65, which resulted in reduced PARP10 expression^34^.

### Positive regulation of NF-κB signaling by ARTD family members

Gemcitabine-resistant prostate carcinoma depends on constitutively active NF-κB signaling and expresses high levels of PARP14 (ARTD8), which in turn correlates with poor patient survival^35^. Downregulation of PARP14 by siRNA decreases NF-κB activation in gemcitabine-resistant prostate carcinoma cells and consequently promotes apoptosis, suggesting that PARP14 is important for cell viability and required for constitutive NF-κB signaling. Mechanistically, decreased NF-κB signaling was explained by a reduced phosphorylation of IκBα^35^. However, the detailed molecular mechanism has not yet been elucidated.

The canonical NF-κB signaling pathway is activated in response to viral infection^30^. Mechanistically, binding of 5′-triphosphate-modified RNA to RIG-I complexes enhances its association with MAVS, which ultimately induces IKK complex activation, leading to the degradation of IκBα and the release of NF-κB dimers^36^. Interestingly, the catalytically inactive RNA binding protein PARP13 (ARTD13)^37^ is able to restrict oncogenic virus replication by stabilizing the binding of activated RIG-I complex to MAVS, leading to increased NF-κB signaling^36^. This suggests that PARP13 might protect cells against malignant transformation and cancer development^38^. However, the exact molecular mechanism of PARP13’s mode of action remains elusive. Although PARP13 was reported to be catalytically inactive, its antiviral function is depended on trans-ADP-ribosylation of PARP13 itself, which is controlled by a so far unknown ART member^38^.

NF-κB signaling upon LPS stimulation of a murine macrophage-like cell line is positively regulated by p62/SQSTM1, whereby the scaffolding protein p62 enhances the TLR4-induced NF-κB signaling^39^. Interestingly, PARP12 localizes to p62/SQSTM1 foci and PARP12 activity is required for the activation of the NF-κB signaling cascade^40^. This observation was further strengthened by the co-localization of PARP12 and TRIF, a downstream component of the TLR4 receptor, required for TLR4 dependent NF-κB activation^40^. However, a molecular mechanism of NF-κB signaling activation by PARP12 remains to be defined. The capacity of PARP12 to promote NF-κB-dependent gene transcription requires the presence of an active catalytic domain. However, as the *K*_m_ NAD^+^ of PARP12 is ~300 µM, this would suggest that the NAD^+^ concentration is higher in p62/SQSTM1 foci compared to the rest of the cytoplasm. Conversely, it might also be possible that the binding of PARP12 to its substrate decreases its *K*_m_ NAD^+^, thereby allowing PARP12 activity.

ER-stress-induced inflammation and activation of the unfolded protein response (UPR) is relayed via ER-associated sensors of ER stress. These sensors are described to substantially contribute to tumor progression and metastasis in a NF-κB-dependent manner^41^. Mechanistically, activated IRE-1α recruits TRAF2, which in turn results in the activation of the canonical pathway through IKK and the release of NF-κB dimers. In parallel, PERK leads to an eIF2α-dependent attenuation of translation and thereby reduces overall IκB levels, shifting the balance from complexed inactive NF-κB toward a free and transiently active NF-κB^42^. Upon ER stress, PARP16, a tail-anchored ER protein, modifies itself as well as two ER stress sensors, IRE-1α, and PERK^43^. Interestingly, the third sensor of ER stress, ATF6, seems not to be regulated by PARP16. PARP16 activation is sufficient to promote IRE-1α and PERK signaling even in the absence of ER stress and is strictly required for their activity during UPR^43^. Since these data suggest a strong correlation between UPR functionality and PARP16 activity, it would be tempting to propose a role for PARP16 in ER-stress-induced inflammation via NF-κB signaling in cancer. In support of this observation, inhibition of PARP16 results in the suppression of ER-stress-induced PERK phosphorylation and increases cancer cell apoptosis under untreated and ER-stress-induced conditions^44^. However, it remains elusive whether the inhibition of PARP16 also interferes with NF-κB activation upon ER-stress and thereby induces apoptosis by inhibition of NF-κB signaling. Intriguingly, the remarkably high *K*_m_ NAD^+^ of PARP16 (582 µM) suggests that PARP16 is not active under basal conditions. It is, however, possible that ER-stress increases the local ER NAD^+^ concentrations to a level that would allow PARP16 activity.

Both the PARP1 (ARTD1) protein as well as its enzymatic activity are associated with increased NF-κB signaling. NF-κB target gene expression in TNFα-stimulated NIH3T3 fibroblasts lacking PARP1 was repressed even though NF-κB was localized to the nucleus^45^. Furthermore, treatment of wild-type or PARP1-deficient mice with lipopolysaccharide (LPS) further supports the notion that PARP1 is involved in the transcriptional activity of NF-κB^45^. Mechanistically, LPS dependent NF-κB activation in primary murine fibroblasts is mediated by the interaction of PARP1 and two transcriptional coactivators, CREB-binding protein (CBP) and p300^46^. The formation of this complex results in PARP1 acetylation, which in turn allows the PARP1-CBP-p300 complex to interact with the p50 subunit of NF-κB. NF-κB signaling is consequently activated and the transcription of proinflammatory cytokines, chemokines, transcription factors, and other inflammatory mediators is initiated^47^. Besides, the PARP1 enzymatic activity has also been suggested to be important for NF-κB signaling^48^. Poly-ADP-ribosylation of p65 was found to be critical for its nuclear retention in TLR4-stimulated smooth muscle cells^48^. PARP1-dependent poly-ADP-ribosylation of p65 reduced the interaction with nuclear exporter Crm1, increasing p65 nuclear retention and ultimately enhancing NF-κB target gene expression^48^. Interestingly, irradiation-induced DNA damage in mice and consequential activation of PARP1 enzymatic activity results in the dynamic assembly of a complex comprising NEMO and likewise induces NF-κB activation^49^. This suggests that PARP1 can activate NF-κB even by endogenous signals to avert programmed cell death and emphasizes the potential beneficial effect of PARP inhibitors in combination with ionizing radiation as a tumor therapy^50^. Variation in nuclear NAD^+^ concentration governs NF-κB expression levels and transcriptional activity^25^. In this regard, the NAD^+^-dependent deacetylase SIRT1 negatively regulates PARP1 by inhibiting the expression of PARP1 and possibly through deacetylation of PARP1, reducing its enzymatic activity^25^. In contrast, since the *K*_m_ NAD^+^ value of PARP1 is lower compared to the one of SIRT1, constitutive active PARP1 inhibits SIRT1 by consuming nuclear NAD^+^, which ultimately represses SIRT1^51^. Therefore, lower local NAD^+^ concentrations favor PARP1’s role as a transcriptional co-activator of NF-κB, while a high NAD^+^ availability promotes SIRT1 inhibition of NF-κB activity through the deacetylation of p65 and reduction of PARP1 levels^25^. Hence, the competition between PARP1 and SIRT1 for NAD^+^ directly links the NF-κB pathway to the metabolic state of a cell.

During non-canonical NF-κB signaling, NF-κB2 processing is regulated by NIK, which in turn is downregulated by the non-canonical IKK TBK1^52,53^. In a NF-κB-independent context, TBK1 is described as an activator of IRF3 in response to viral infection^54^. Of note, TBK1 kinase activity and thus the efficacy of the antiviral response is negatively regulated by PARP7 (ARTD14/TIPARP) mediated ADP-ribosylation^55^. However, whether there is a direct link between PARP7 and the downstream activation of NF-κB, through the inhibition of TBK1, remains elusive.

### Negative regulation of NF-κB signaling by ARTD family members

In HeLa and U2OS cells, activation of the IKK complex and thus NF-κB signaling is inhibited by PARP10^56^. Mechanistically, PARP10 decreases K63-linked polyubiquitination on NEMO, which represses IKK assembly and activation^56^. Although NEMO is found to be ADP-ribosylated by PARP10 in vitro, neither the overexpression of a catalytic mutant or a ubiquitin-binding deficient mutant represses the inhibitory effect of PARP10 toward NF-κB in cells^56^. This suggests that an unknown protein property of PARP10 is involved in NEMO inhibition. Furthermore, in hepatocellular carcinoma, the inhibition of NEMO ubiquitination by PARP10 is negatively regulated by PLK1-mediated phosphorylation of PARP10. In this regard, PARP10 inhibition increases NF-κB signaling by releasing PARP10’s repression of NEMO^34^. Interestingly, PLK1 is mono-ADP-ribosylated by PARP10, which significantly inhibits its kinase activity and oncogenic function^34^. In addition, PARP10 dependent mono-ADP-ribosylation of GSK3β, a kinase that promotes NEMO stability and enhances NF-κB signaling^57^, represses its kinase activity in vitro^58^. In an active state, GSK3β positively regulates SCF^Fbw7^ activity and thereby promotes the degradation of the p100, ultimately releasing the repression of NF-κB1 and promoting canonical NF-κB signaling^59^. Together, these findings support the notion of PARP10 as a negative regulator of NF-κB via inhibition of GSK3β activity. However, the influence of PARP10 on GSK3β and thus on NF-κB activation requires further evaluation, since mono-ADP-ribosylation of GSK3β only mildly reduced its kinase activity in vitro^58^. Nevertheless, this proposed mode of action is strengthened by the fact that removal of PARP10-mediated mono-ADP-ribosylation on GSK3β enhances its kinase activity^60^.

In NF-κB signaling, PARP1 is not only described as a transcriptional co-factor but, for some genes, also as a transcriptional repressor^61^. The expression of a subset of NF-κB target genes in murine macrophages is repressed by PARP1 under basal conditions and enhanced upon activation of the NLRP3 inflammasome and caspases 1/7^62^. Caspase-dependent cleavage of PARP1 results in its release from chromatin, which promotes local chromatin de-condensation and allows enhanced gene expression^63^.

## Interplay between JAK/STAT signaling and ARTD family members

### Overview of the JAK/STAT pathway

**Fig. 2:**
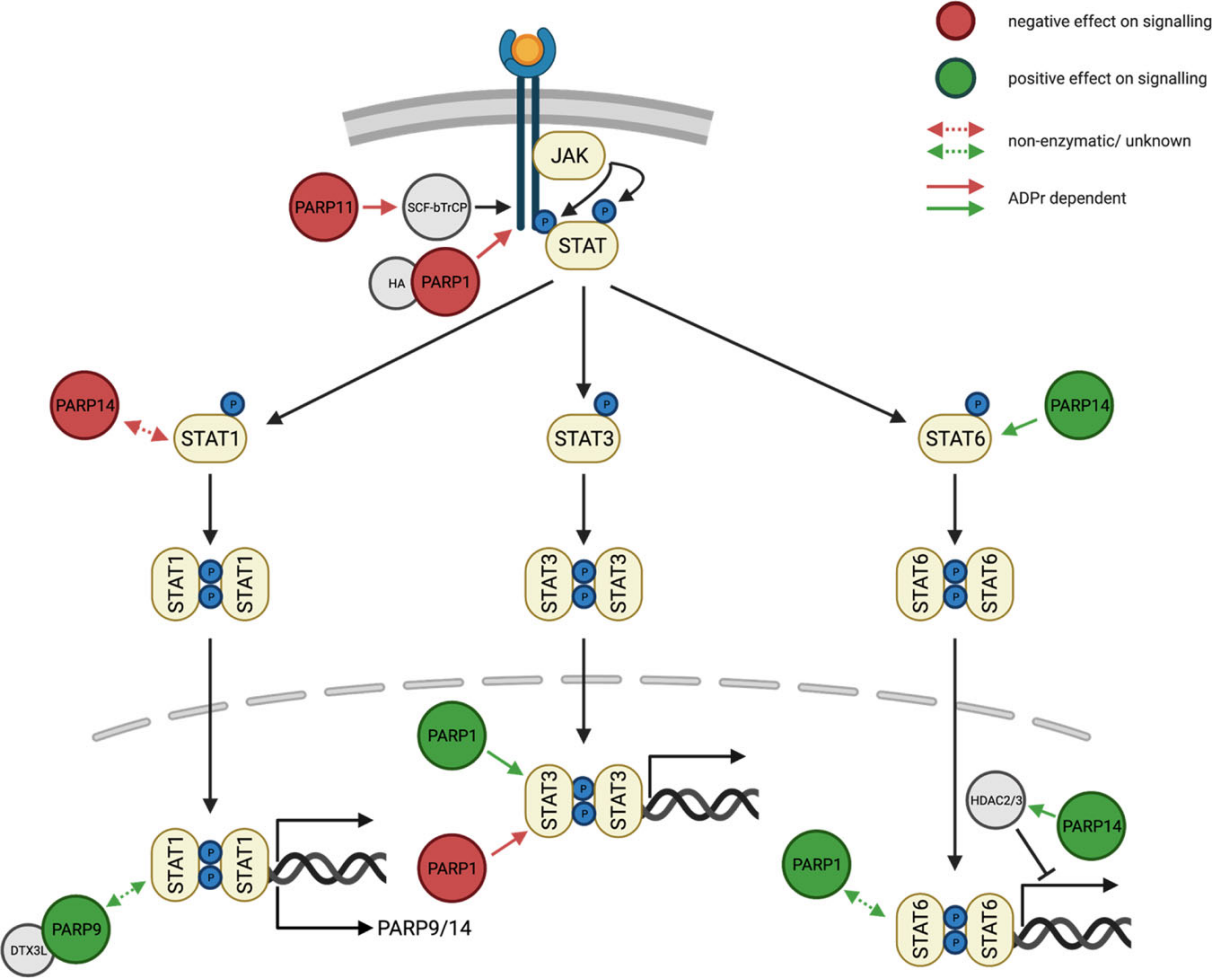
Schematic overview of the interplay between JAK/STAT signaling and ARTD family members. Positive regulations of JAK/STAT signaling by ARTD family members are depicted in green, while negative effects of ARTDs on the signaling pathway are shown in red. Solid lines indicate the contribution of ADP-ribosylation to the regulation of JAK/STAT signaling. In case the contribution of ADP-ribosylation was not described or the protein itself rather than its enzymatic activity is involved in the regulation, the interactions are represented by dashed lines.

The Janus kinase/signal transducer and activator of transcription proteins (JAK/STAT) signaling pathway, known to operate in response to over 50 cytokines and growth factors, is a central communication node for the immune system^64^. On the molecular level, the pathway consists of the cytoplasmatic kinase JAK, which directly interacts with a variety of transmembrane receptors and the transcription factor STAT (Fig. 2). In mammals, 4 JAKs (JAK1, JAK2, JAK3, TYK2) and 7 STATs (STAT1, STAT2, STAT3, STAT4, STAT5a, STAT5b, STAT6) are activated by cytokine receptor oligomerization^64^. Extracellular association of cytokines with their corresponding transmembrane receptors and receptor oligomerization provokes the trans-activation of JAKs, which subsequently phosphorylate the cytoplasmic tails of the receptors to create the requisite docking sites for STATs. This puts JAKs and STATs in spatial proximity and allows JAKs to tyrosine-phosphorylate STATs, which results in STAT dimerization, nuclear translocation, DNA binding and, ultimately, the induction of gene transcription^65^. Each cytokine or receptor is typically associated with a particular STAT. However, it was recently found that most cytokines engage more than one STAT member, leading to the formation of not only STAT homodimers but also heterodimers and higher-order tetramers^64^. Activation of JAK/STAT signaling conveys a large variety of transcriptional outputs^66^. The specificity of this pathway is regulated by (i) cell lineage-specific susceptibility toward STAT activation, (ii) qualitative differences in the duration and/or intensity of the downstream STAT signaling, and (iii) quantitative expression differences between co-activated STATs^67^. Although the canonical JAK/STAT pathway is simple and direct, pathway components regulate and/or are regulated by members of other signaling pathways, including signaling cascades involving the kinases ERK and PI3K^68^.

Over the past 20 years, the link between JAK/STAT signaling and oncogenesis has become a major subject in cancer biology. It has long been known that STAT hyperactivity can drive cellular transformation downstream of classic oncogenes^68^. This hyperactivity, which typically involves STAT3 and/or STAT5, is now considered a defining characteristic of most solid and blood cancers^69^.

### Influence of JAK/STAT signaling on ARTD family member expression

A proteomic study conducted in IFNγ-stimulated THP-1 cells revealed that PARP9 (ARTD9) and PARP14 were highly expressed and increasingly ADP-ribosylated^70^. Interestingly, in macrophages PARP9 and PARP14 exert anti- and proinflammatory effects respectively, and thereby regulate macrophage activation^71^. However, whether these findings are dependent on their enzymatic activity is currently under debate and will be discussed in greater detail in the section “Positive regulation of JAK/STAT signaling by ARTD family members”. In addition, the expression of PARP9 is regulated by the IFNγ-JAK2/STAT1-IRF1 signaling axis that is required for cancer cell survival in diffuse large B-cell lymphoma (DLBCL) with an active host inflammatory response^72^.

### Positive regulation of JAK/STAT signaling by ARTD family members

In macrophages, IL-4-induced STAT6 phosphorylation and the subsequent expression of anti-inflammatory genes are dependent on PARP14 activation and seem to include PARP14-mediated STAT6 ADP-ribosylation^71,73^. This finding is based on the assumption that the proposed STAT6 ADP-ribosylation site lies in close proximity to a potentially functional relevant STAT6 phosphorylation site (Tyr629) and that PARP14-mediated ADP-ribosylation would promote phosphorylation-dependent activation of STAT6. However, this interpretation was called into question, since the proposed STAT6 phosphorylation site (Tyr629) was so far not described. In this regard, a large body of research agreed on Tyr641 as the functionally critical and possibly sole phosphorylated tyrosine of human STAT6^74^. Nonetheless, phosphorylation and ADP-ribosylation sites, specifically for Ser and Tyr residues, significantly overlap, suggesting a site-specific regulatory interplay between the two modifications^75,76^. Interestingly, Ser phosphorylation and ADP-ribosylation seem to be mutually exclusive^77^. The emerging crosstalk between ADP-ribosylation and phosphorylation might represent an additional layer of regulation which requires further functional validation. Although direct ADP-ribosylation of STAT6 by PARP14 remains elusive^74^, it was reported for the mouse lymphoma cell line M12 B that the catalytic activity of PARP14 functions as a molecular switch for IL-4-induced and STAT6-dependent gene expression of IL-4 responsive promotors^78,79^. In presence of IL-4, PARP14 promotes the mono-ADP-ribosylation of HDAC2 and HDAC3 which leads to their dissociation from the IL-4 responsive promotors and facilitates promotor-binding of STAT6 as well as transcriptional co-factors such as p300, subsequently resulting in active gene transcription^80^. Furthermore, IL-4 and STAT6-dependent Th2 responses and immunoglobulin class switching to IgE are collectively linked to the development of asthmatic conditions^81^. Consequently, the functional relevance of PARP14-mediated ADP-ribosylation for STAT6-dependent gene activation might be a potential therapeutic target in order to reduce the progression of allergic airway diseases.

Interestingly, PARP1 positively regulates STAT6-dependent transcriptional activation^82^. PARP1 downregulation results in decreased STAT6 protein stability, which in turn is reflected by a reduction in IL-5 expression in murine splenocytes^82^. Furthermore, depletion of NAD^+^ via excessive PARP1 activation, is considered to contribute to the pathogenesis of various cardiovascular diseases, including atherosclerosis and cardiac hypertrophy^83^. In a phenylephrine-induced cardiac hypertrophy model, PARP1 and its enzymatic activity retain phosphorylated STAT3 in the nucleus. This prolongs STAT3 transcriptional activity independent of JAK2 activity and contributes to the hypertrophy phenotype^84^. A detailed molecular mechanism of how PARP1 affects the phosphorylation status of STAT3 was not described. Moreover, either PARP1 protein or its enzymatic function regulates the expression of multiple pro- and anti-inflammatory cytokines, which in turn modulate the activity of the JAK/STAT signaling cascade in a para- or autocrine fashion. The role of PARP1 in cytokine expression was already extensively discussed elsewhere^37,82,85^.

PARP9 and its binding partner DTX3L are associated with STAT1 and are important for interferon signal transduction^86^. Noteworthy, the PARP9/DTX3L complex acts as the rate limiting factor for STAT1 transcriptional activity and is able to enhance the host defense response following viral infection^86^. In support of this observation, PARP9 activates proinflammatory genes and STAT1 phosphorylation in response to IFNγ in monocytes^73^. Although PARP9 was thought to be enzymatically inactive, PARP9 as a heterodimer with DTX3L seems to possess mono-ADP-ribosylation activity^87^. Therefore, in the presence of high NAD^+^ concentrations PARP9 modifies ubiquitin and thereby restrains DTX3L’s E3 activity. Remarkably, in DLBCL PARP9 stimulates the phosphorylation of both STAT1 isoforms. However, it especially promotes the nuclear accumulation of the transcriptionally repressive isoform STAT1β^88^. In this regard, PARP9 directly inhibits, together with STAT1β, the expression of tumor suppressor IRF1. Conversely, PARP9 enhances the expression of the proto-oncogenes IRF2 and BCL6. Overall, PARP9 represses the anti-proliferative and pro-apoptotic IFNγ-STAT1-IRF1-p53 axis and mediates proliferation and survival in DLBCL^88^.

### Negative regulation of JAK/STAT signaling by ARTD family members

Type 1 interferon receptor (IFNAR1) protein stability is an essential determinant of the IFN1 antiviral response. Strikingly, viral infection promotes the expression of PARP11 (ARTD11), which in turn mono-ADP-ribosylates the ubiquitin E3 ligase β-TrCP. This ultimately results in IFNAR1 ubiquitination and degradation, and finally in the suppression of the IFN antiviral signaling^89^.

Similarly, influenza A viral infection results in the transient cytoplasmic co-localization of HA and PARP1, which in turn mediates IFNAR1 degradation in an ADP-ribosylation-dependent manner^90^. However, the molecular mechanism underlying the PARP1-dependent IFNAR1 degradation has yet to be investigated.

In IFNγ-stimulated monocytes, PARP14 suppresses proinflammatory genes and STAT1 phosphorylation^73^. However, it was called into question whether this finding is dependent on the catalytic activity of PARP14. This is mostly because a previously described SUMO conjugation site of STAT1, which is a critical regulator of IFNγ signaling and in close proximity to the proposed ADP-ribosylation site of STAT1, was not further considered^74^.

In ovarian, lung, and colon cancer cell lines, PARP1 downregulates PD-L1 expression by ADP-ribosylating STAT3 and, thus, decreasing its phosphorylation^91^. This contrasts with the positive regulation of STAT3 discussed in the “Positive regulation of JAK/STAT signaling by ARTD family members” section and emphasizes the context-specific regulation of STAT3 by PARP1.

## Interplay between Wnt/β-catenin signaling and ARTD family members

### Overview of the canonical Wnt/β-catenin signaling pathway

**Fig. 3:**
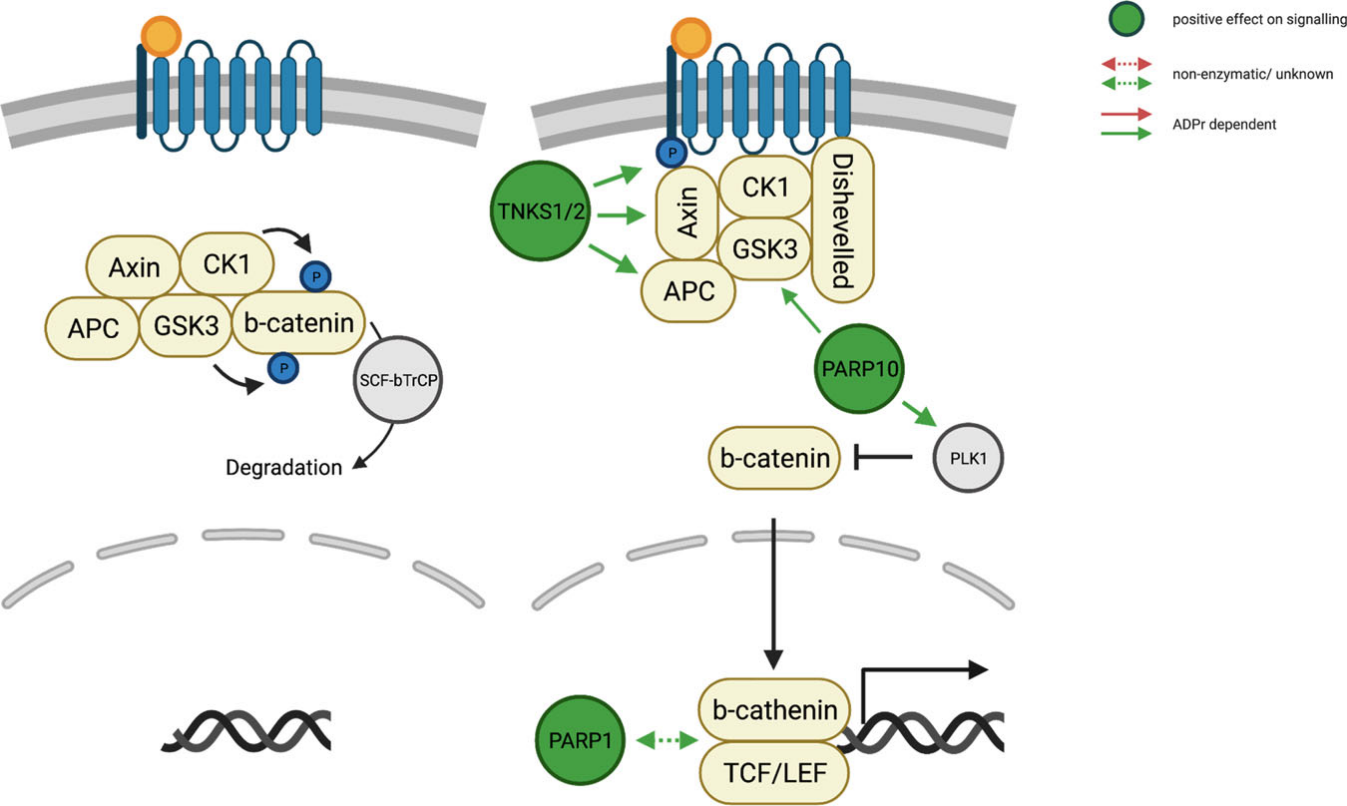
Schematic overview of the interplay between Wnt/β-catenin signaling and ARTD family members. Uninduced (left panel) and induced (right panel) Wnt/β-catenin signaling. Positive regulations of Wnt/β-catenin signaling by ARTD family members are depicted in green. Solid lines indicate the contribution of ADP-ribosylation to the regulation of Wnt/β-catenin signaling. In case the contribution of ADP-ribosylation was not described or the protein itself rather than its enzymatic activity is involved in the regulation, the interactions are represented by dashed lines. (TNKS1/2, Tankyrase1/2).

Signaling induction via the family of Wnt secreted glycolipoproteins either in an auto- or paracrine fashion is one of the fundamental mechanisms that direct cell proliferation, cell polarity, and cell fate determination during embryonic development and tissue homeostasis^92^. In the absence of Wnt ligands the plakoglobin β-catenin, also critical for cell–cell adhesion, is sequestered in the cytoplasm by the Axin complex that comprises the scaffolding protein Axin, the tumor suppressor APC and the two kinases CK1 and GSK3^93^. In this steady-state, CK1 and GSK3 sequentially phosphorylate β-catenin, resulting in β-catenin ubiquitination mediated proteasomal degradation by SCF^β-TRCP^, thus preventing its nuclear translocation (Fig. 3)^93^. Extracellular binding of Wnt ligands to the Frizzled receptor and its co-receptors LRP6 or LRP5 recruits the scaffolding protein Dishevelled to the cytoplasmic domain of the Frizzle receptor^93^. The newly formed receptor complex recruits CK1 and GSK3 and thus facilitates the phosphorylation of the co-receptors LRP6/5, which in turn recruits Axin. Binding of Axin to the plasma membrane prevents phosphorylation of β-catenin and shifts the balance toward unmodified and free β-catenin. Ultimately, this promotes its nuclear accumulation and subsequently complex formation with TCF/LEF to induce gene expression of Wnt target genes (Fig. 3)^94^.

Somatic and germline mutations promoting constitutive Wnt/β-catenin signaling often correlate with the onset of tumorigenesis^95^. The functional consequences of aberrant Wnt/β-catenin signaling include an increase in proliferation, migration, and the promotion of an epithelial to mesenchymal transition^95^. Furthermore, constitutive activation of Wnt/β-catenin in cancer cells promotes aerobic glycolysis by transcriptionally suppressing an integral enzyme of the mitochondrial respiratory chain^96^. This Wnt/β-catenin-dependent metabolic switch could ultimately increase free cytoplasmic NAD^+^ levels.

### Influence of Wnt signaling on ARTD family member expression

So far, Wnt/β-catenin has not been described to regulate the expression of the known ARTD family members.

### Positive regulation of Wnt signaling by ARTD family members

Tankyrase 1 and Tankyrase 2 (PARP5a/b or ARTD5/6) regulate telomere length^97^, centrosome maturation^98^, proteasome assembly^99^, and establishment of the mitotic spindle^100^. Intriguingly, β-catenin stability, and thus active Wnt signaling, seem to be regulated by Tankyrase 1/2 since the double knock out Tankyrase 1/2 causes early embryonic lethality in mice^101^. In this regard, inhibition of Tankyrase 1/2 destabilizes β-catenin by enhancing the stability of Axin, which subsequently reduces the growth of β-catenin-dependent colorectal cancer cells^101^. On the molecular level, Tankyrase 1/2 dependent ADP-ribosylation induces ubiquitination-dependent degradation of Axin. Based on a siRNA screen in HEK293 cells, RNF146, a RING-domain E3 ubiquitin ligase, was identified as a positive regulator of Wnt signaling^102^. Mechanistically, RNF146 interacts through its WWE domain with poly/oligo-ADP-ribosylated Axin resulting in the degradation of modified Axin^102^. This provides the first line of evidence for crosstalk between ADP-ribosylation and ubiquitination. Interestingly, this crosstalk not only applies to the Wnt signaling pathway and Tankyrases but seems to be a more general mechanism in regulating protein stability. For example, auto-PARylation of PARP1 recruits TRIP12, which catalyzes the ubiquitination of PARP1 and its degradation^103^. Similarly, HUWE1 promotes the degradation of auto-MARylated PARP7^104^, emphasizing the functional importance of PTM interplay.

Tankyrase inhibitors were previously thought to disrupt Wnt signaling solely by increasing the basal levels of Axin and subsequently increasing β-catenin degradation^105^. However, the degree to which the basal level of Axin increased following Tankyrase 1/2 inhibition was not sufficient to disrupt Wnt signaling in *Drosophila melanogaster*^105^. Therefore, it was suggested that the *Drosophila melanogaster* Tankyrase 1/2 homolog promotes the interaction of Axin with phospho-LRP6. It was suggested that the Tankyrase 1/2 homolog in *Drosophila melanogaster* acts in a biphasic manner: (i) at the basal level it promotes the degradation of Axin, which alone is not sufficient to induce Wnt signaling; (ii) upon binding of Wnt ligands to the receptor, Tankyrase 1/2 activity is enhanced and induces Axin binding to phosphor-LRP6, which shifts the balance toward stable β-catenin^105^. The mechanism of Wnt ligand-dependent activation of Tankyrase 1/2 has yet to be defined.

The promotion of apoptosis or tumor regression in colon and lung cancer after treatment with Tankyrases inhibitors, suggests a role of Tankyrase 1/2 as oncogenic factors^106–109^. Colon cancers often harbor tumor suppressor APC inactivating mutations that lead to constitutive activation of Wnt signaling^110^. Surprisingly, even under these conditions, Tankyrase 1/2 inhibition reduces the cellular β-catenin levels, which could be explained by Tankyrase 1/2 modifying and inhibiting APC2 and thus decreasing the activity of the degradome^111^. However, the mechanisms of APC2 regulation by Tankyrase 1/2 appears to be different compared to Axin, as Tankyrase 1/2 inhibition did not result in global changes of APC2 protein levels. This suggests that Tankyrase 1/2 promote Wnt/β-catenin signaling by an additional mechanism that has not yet been characterized.

Since Axin is still degraded after extended Wnt signaling in a Tankyrase 1/2-independent fashion, Tankyrase 1/2 are not solely responsible for the Axin degradation. In support of this notion, *Drosophila melanogaster* lacking Tankyrase 1/2 homolog are viable and fertile, and mice lacking both Tankyrase 1 and Tankyrase 2 proteins, although embryonic lethal, survive to E10 without obvious defects in Wnt signaling. Thus, while Tankyrase 1/2 can fine-tune the Wnt signaling pathway it seems not to be an essential regulator^112^.

The kinase activity of GSK3 is a crucial repressor of the β-catenin stability and is negatively regulated by PARP10-mediated mono-ADP-ribosylation, which leads to increased β-catenin levels^58^. This proposed mode of action was further strengthened by the discovery that the hydrolase MacroD2 is able to counteract PARP10-mediated mono-ADP-ribosylation of GSK3, which restores its kinase activity^60^. Noteworthy, MacroD2 knockdown in HCC cells markedly enhanced proliferation and invasiveness in vitro, tumor progression in vivo, and promoted epithelial–mesenchymal transition^113^. Furthermore, PLK1 induces the destabilization of β-catenin^114,115^ and is mono-ADP-ribosylated by PARP10^34^. The modification of PLK1 significantly reduces its enzymatic activity^34^, suggesting a potential secondary mechanism by which PARP10 might positively regulate β-catenin stability.

Wnt target gene expression in APC deficient family and sporadic colorectal cancer (CRC) is enhanced by PARP1 as a co-factor of TCF-4/β-catenin^61^. Conversely, Ku70 has been observed to associate with TCF-4/β-catenin and to repress TCF/LEF function^116^. Noteworthy, Ku70 competes with PARP1 for binding to the complex, suggesting that this mutually exclusive behavior is a determinant for Wnt target gene expression. In colon cancer, PARP1 is often overexpressed, which indicates its beneficial functional role for β-catenin transcriptional activity^116^.

As mentioned above, constitutive Wnt/β-catenin signaling in cancer cells can promote the metabolic switch from oxidative phosphorylation to aerobic glycolysis^96^. This transition results in the increase of cytoplasmic NAD^+^ levels^117^. In this context, the expression of LDH is increased and thus favors NADH oxidation and increases cytoplasmic NAD^+^ levels^118^. Therefore, it would be interesting to elucidate whether increased Wnt/β-catenin signaling promotes a positive feedback loop involving increased free cytosolic NAD^+^ and enhanced ADP-ribosylation.

### Negative regulation of Wnt signaling by ARTD family members

So far, no negative regulation of the Wnt signaling by ARTD family members was reported.

## Interplay between MAPK signaling and ARTD family members

### Overview of the MAPK pathway

**Fig. 4:**
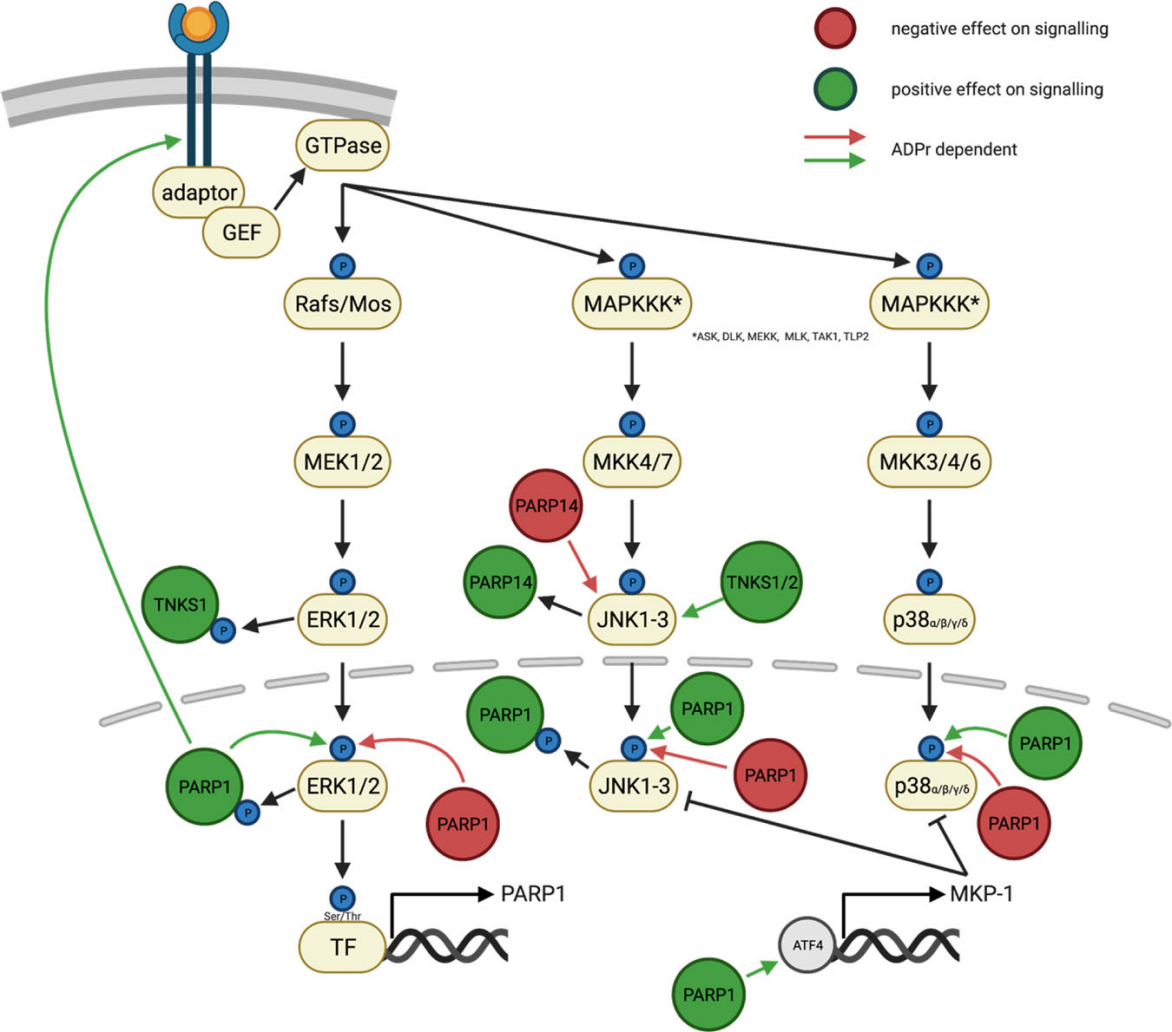
Schematic overview of the interplay between MAPK signaling and ARTD family members. Positive regulations of MAPK signaling by ARTD family members are depicted in green, while negative effects of ARTDs on the signaling pathway are shown in red. Solid lines indicate the contribution of ADP-ribosylation to the regulation of MAPK signaling (TNKS1/2, Tankyrase1/2; Ser, Serine; Thr, Threonine).

The mitogen-activated protein kinases (MAPK) pathway is a cascade of cytoplasmic phosphorylation events, initiated by the binding of different ligands (i.e., mitogens, growth factors, and cytokines) to their respective receptors^119–121^. The phosphorylation cascade comprises three sequentially activated protein kinases starting with the MAPK kinase kinases (MKKKs) that are followed by the MAPK kinases (MKK) and the MAPKs (Fig. 4)^119–121^. The MAPKs are thus the effector kinases of the pathway that catalyze phosphorylation of Ser and Thr residues of target proteins. Phosphorylation of target proteins modulates their enzymatic activity, subcellular localization, or capacity to engage in interactions with other proteins^119–121^. MAPKs are classified into distinct subgroups, which comprise the extracellular-signal-regulated kinases (ERKs), c-Jun N-terminal kinase (JNK), and the p38 kinases^119–121^.

In general, differences in signal-duration and ERK1/2 organelle localization modulate the outcome of ERK signaling^122^. While growth factor and mitogen-activated ERK signaling is mainly involved in promoting cell growth, proliferation, and survival^123^, prolonged retention of activated ERK1/2 in the nucleus in neuronal cells resulted in cell death^124^. The stress-activated JNK can be activated as a consequence of inflammatory cytokine signaling or environmental stress and is important for a proper immune response and cytokine production as well as stress-induced apoptosis and cell proliferation^123^. In cancer cells, JNK regulates opposing cell fates ranging from inducing apoptosis to facilitating proliferation and survival^125^. Finally, the p38 kinase pathway can be activated by cytokines, TLR ligands, growth factors, and environmental stress and is involved in the production of inflammatory mediators^123^. Besides its involvement in inflammation, p38 was described to be associated with various phenotypes ranging from cell growth and cell differentiation to cell death^123^. In cancer cells active ERK1/2 signaling promotes the expression of transcriptional regulators that in turn drive the expression of glycolytic enzymes^126^. Moreover, JNK2, but not JNK1, promotes aerobic glycolysis by reducing the phosphorylation of PKM2 (see the “Negative regulation of MAPK signaling by ARTD family members” section)^126^.

### Influence of MAPK signaling on ARTD family member expression and activity

ERK signaling positively regulates both PARP1 expression and activity^127,128^. Inhibition of MEK, the MAPK kinase upstream of ERK, in conditioned medium-stimulated endothelial cells led to downregulation of PARP1 expression^127^, while overexpression of p-ERK2 in neurons led to an increased PARP1 activity^129^. In turn, the p-ERK2-induced PARP1 activation correlated with enhanced phosphorylation of ERK targets^129^. A positive correlation between ERK signaling and PARP1 activity was also observed in astrocytes, where both ERK1 and ERK2 phosphorylated PARP1^130^. However, only ERK2 was essential for PARP1 activation^130^. In addition, following prolonged seizure in rats the enzymatic activity of PARP1 in astrocytes was reduced after ERK inhibition^131^, indicating that ERK might be a positive upstream regulator of PARP1 signaling. Moreover, MEK inhibitors markedly decreased MNNG-induced PARP1 activation and consequential astrocyte cell death^130^. A positive regulatory loop between PARP1 and ERK was proposed, whereby p-ERK-induced PARP1 auto-PARylation promoted PARP1-dependent retention of p-ERK in the nucleus, and ultimately facilitated phosphorylation of nuclear targets by p-ERK^127,132^. The interaction between p-ERK and activated PARP1 was reviewed in greater detail elsewhere^128,132^. Of note, MNNG-induced PARylation in HeLa cells was not affected by MEK inhibitor treatment^133^, suggesting that ERK affects the activity of ARTDs in a cell-type-specific manner.

Besides PARP1, Tankyrase 1 activity was also found to be modulated by ERK signaling^134^. In insulin, PDGF and EGF-stimulated 3T3-L1 fibroblasts and adipocytes ERK phosphorylated Tankyrase 1^134^. ERK-dependent phosphorylation increased Tankyrase 1 auto-modification and ADP-ribosylation of its binding partner IRAP (an abundant protein in GLUT4 storage vesicles) in vitro. Conversely, after insulin stimulation, endogenous Tankyrase 1 was not ADP-ribosylated^134^. Intriguingly, the enzymatic activity of Tankyrase 1 seems to be essential for GLUT4 storage vesicle trafficking and thus insulin-stimulated glucose uptake^135^. Of note, ERK-dependent Tankyrase 1 phosphorylation was also found in a different context, namely upon herpes simplex virus (HSV) infection of Hep-2 cells^136^. Moreover, Tankyrase 1 depletion or inhibition by Tankyrase inhibitor impaired HSV growth, suggesting that the enzymatic activity, potentially enhanced by phosphorylation, of Tankyrase 1 may be essential for HSV infection^136^.

In addition, JNK1 was postulated to be a positive upstream regulator of PARP1 activation in H_2_O_2_-mediated cell death in mouse embryonic fibroblasts (MEFs)^137^. JNK1 sustained PARP1 activation only upon prolonged H_2_O_2_ stimulation by directly phosphorylating PARP1, but it did not affect ADP-ribosylation during early timepoints^137^. Similarly, in the context of multiple myeloma, JNK2 positively regulated PARP14 protein levels through an unknown mechanism^138^.

### Positive regulation of MAPK signaling by ARTD family members

The MAPK signaling pathways were described to be positively regulated by PARP1 and Tankyrase 1/2. Under normal conditions, MEK1/2 (MAPKK)-mediated phosphorylation of ERK1/2 (p-ERK1/2) induces conformational changes in ERK1/2 that result in ERK1/2 activation^120,122,139^. This ultimately induces the phosphorylation of downstream substrates that promote cell growth, survival, and migration^120,122,139^. Interestingly, PARP1-mediated ADP-ribosylation was found to promote cell survival by enhancing ERK phosphorylation (p-ERK) in different cellular contexts^127,140–142^. Knockdown and/or inhibition of PARP1 in lung cancer and osteosarcoma cells decreased ERK phosphorylation, which reduced cell proliferation and migration, and increased apoptosis^141,142^. In lung cancer cells, not only ERK phosphorylation but also total ERK protein levels were decreased upon inhibition of ADP-ribosylation or PARP1-siRNA treatment^141^. Interestingly, PARP1 inhibition or knockdown also reduced the expression and phosphorylation of EGFR, an upstream activator of ERK^141^. This suggests that PARP1-mediated ADP-ribosylation not only reinforces ERK1/2 signaling but also the expression of its pathway components. Conversely, siRNA-mediated knockdown of PARP1 in osteosarcoma cells did not affect the abundance of total ERK^142^. In addition, the positive effect of ADP-ribosylation on ERK signaling and survival was also observed in a non-cancer cell context^127,140^. Inhibition of ADP-ribosylation (PJ-34) in primary epithelial cells stimulated by conditioned medium not only reduced ERK phosphorylation, but also phosphorylation of its downstream target Elk-1^127^. Consequently, cell viability and migration were reduced^127^. A reduction in ERK activation in response to inhibition of ADP-ribosylation was also observed in macrophages^140^. LPS-induced ERK phosphorylation was reduced by the treatment with PARP inhibitors (NA, MIGB)^140^. Together, these findings indicate an involvement of PARP1 and ADP-ribosylation in survival, proliferation, and growth by increasing ERK phosphorylation, which modulates ERK pathway activity. While most studies did not investigate the mechanism of PARP1-dependent modulation of ERK activity, one proposed that auto-PARylated, nuclear PARP1 acts as a scaffold protein that retains p-ERK in the nucleus and allows enhanced downstream phosphorylation^127^.

In the above-mentioned findings, PARP1 activity correlated with enhanced ERK signaling and improved survival. However, under certain circumstances, the inhibition/absence of ADP-ribosylation and the associated reduction in MAPK signaling conveys a protective effect to the cells^143–145^. Perfusion of rat hearts with a cytostatic agent led to an increase in cardiotoxicity and ERK, JNK, and p38 phosphorylation^143^. MAPK phosphorylation was significantly reduced when inhibiting (presumably) PARP1-dependent ADP-ribosylation with BGP-15^143^. Similarly, in LPS-treated mice, ADP-ribosylation inhibition with 4-HQN significantly decreased LPS-induced mortality^145^. Interestingly, the decrease in mortality was partly attributed to tissue-specific reduction of ERK1/2 and p38 phosphorylation^145^. While it is possible that reducing extended ERK phosphorylation has beneficial effects for the cells, it seems more likely that the protective effect observed upon PARP inhibition is associated with the reduction in JNK and p38 activity, both widely described to induce cell death. Even though no mechanism was defined, such a protective role of ADP-ribosylation inhibitors has been observed in a rat model of retinal degeneration, where phosphorylation of JNK and p38 was significantly reduced by PARP inhibitor HO3089^146^. Nonetheless, whether changes in one, two, or all three MAP kinases are the cause of the observed cytoprotective effects of PARP inhibitors and to which extent the cell type and the kind of stimulation play a role remains to be further explored. One could, however, speculate that while moderate activation of PARP1 allows the formation of PAR chains that act as scaffold for p-ERK and enhance downstream signaling^127^, the excessive activation of PARP1 leads to NAD^+^ depletion which in turn might prompt energy sensors to activate JNK or p38 and induce cell death^147^. It is also possible that direct PARylation of a kinase modulates protein-protein interactions and thereby enhances signaling or prompts cell death.

Intriguingly, besides its effect on the MAPK pathway, 4-HQN also decreased the LPS-induced activation of nuclear transcription factor NF-κB, which is presumably activated by members of the MAPK pathway^145^. Moreover, PARP inhibitor-induced AKT signaling has been speculated to mediate the inhibition of MAP kinases^145^, highlighting the possibility of crosstalk between different signaling pathways. The importance of crosstalk and their highly context-specific outcomes can be appreciated by taking into consideration a model of H_2_O_2_-induced cell death in MEFs^144^, another example where inhibition of PARP1 conveys a protective effect to cells. H_2_O_2_ treatment of MEFs resulted in decreased cell survival and rapid ERK phosphorylation compared to the same treatment in PARP1 knockout cells^144^. Survival of WT cells could not be rescued with MEK inhibitors^144^, suggesting that ERK phosphorylation, especially at early timepoints, depends on PARP1 but that ERK signaling does not regulate cell death in this context. On the other hand, AKT phosphorylation kinetics were comparable to the observed ERK activation, and treatment with a PI3K inhibitor not only reduced AKT and ERK phosphorylation, but also increased cell survival, in WT but not PARP1 knockout cells^144^. This suggests that within this context, AKT signaling is the essential pathway for cell survival and ERK activation might merely be a downstream byproduct of AKT signaling. Overall, while PARP1 and ADP-ribosylation correlate with ERK signaling in diverse biological systems, the consequence of PARP1 and ADP-ribosylation-induced ERK phosphorylation is highly cell type and context-specific; under certain circumstances, PARP1-dependent ERK signaling was demonstrated to be beneficial for cell growth, survival and migration. In contrast, in systems where PARP1-dependent cell death was inhibited or PARP1 was absent, the concomitant decline in ERK signaling was shown to be cytoprotective. It is, however, likely that reduced ERK phosphorylation is not the driving force for cell survival in these systems.

Besides its effect on ERK phosphorylation, PARP1 activation also correlates with JNK and p38 signaling in various cell types. This is primarily the case in the context of reactive oxygen species (ROS) dependent PARP1 activation and cell death which depends on JNK and/or p38 signaling^147–149^. H_2_O_2_-induced ADP-ribosylation, cell death, and mitochondrial dysfunction in human WRL-68 cells promoted JNK1/2 and p38 phosphorylation and activation in a PARP1-dependent manner^148,149^. Similarly, cell death during osteo-differentiation depended on ROS-activated PARP1, which in turn promoted p38 phosphorylation resulting in a metabolic collapse and cell death^150^. Furthermore, ROS produced upon hypoxia/reoxygenation of the eye of rats led to PARP1 activation^151^. Inhibition of ADP-ribosylation reversed the induced histological changes in the eye and reduced JNK and p38 phosphorylation^151^. Mechanistically, H_2_O_2_- or ROS-activated PARP1 indirectly upregulated JNK and p38 MAP kinase activities by ADP-ribosylating the activating transcription factor 4 (ATF4) in rats and human cells. Therefore, the binding of ATF4 to its DNA response element was reduced. As a result, the expression of *MKP-1*, a negative regulator of JNK and p38^152^, was decreased, ultimately leading to increased JNK and p38 kinase activity^148,149,151^.

In addition to ROS, MNNG-induced DNA damage can also activate PARP1 and consequently JNK^147^. PARP1-dependent cell death upon MNNG treatment of MEFs required JNK1 activity^147^. Moreover, JNK1 activation was dependent on TRAF2 and RIP1, two proteins involved in tumor necrosis factor receptor 1 (TNFR1)-mediated signal transduction^147,153^. It is possible that NAD^+^ depletion upon PARP1 activation, rather than direct ADP-ribosylation of JNK, modulates the activation status of the TRAF2/RIP1-JNK signaling cascade^147^. Furthermore, PARP1-dependent JNK1 activation was essential for TNFα/ATRA-induced apoptosis in NF-κB repressed human leukemia cells, further highlighting the interplay between TNFR and PARP1/JNK1-mediated cell death^154^. Of note, the involvement of RIP1 in PARP1/JNK-induced cell death is controversial, as not all studies could confirm the indispensable role of RIP1 in this process^155,156^. Overall, PARP1-dependent JNK and p38 phosphorylation is essential for PARP1-induced cell death in different cell types.

Last, Tankyrase 1/2 have been described in JNK activation, albeit in *Drosophila melanogaster* and not mammalian cells^157,158^. Strong activation of JNK caused defective eye and wing development^157,158^. Overexpression of the *Drosophila melanogaster* Tankyrase 1/2 homolog in the wing not only impaired wing formation but also resulted in high activity of caspase 3, suggesting that the apoptotic cell death is induced by JNK signaling^158^. Inhibiting JNK signaling in wings that overexpressed Tankyrase 1/2 decreased the apoptotic cell death, suggesting that JNK is a mediator of *Drosophila melanogaster* Tankyrase 1/2-induced cell death^158^. Tankyrase 1/2-induced JNK activation was dependent on ADP-ribosylation of *Drosophila melanogaster* JNK which induces K63-linked ubiquitination, ultimately enhancing JNK kinase activity^157^.

### Negative regulation of MAPK signaling by ARTD family members

In contrast to the positive correlation between ARTD activation and MAP kinase signaling discussed previously, PARP1- and PARP14-dependent ADP-ribosylation have also been associated with decreased MAPK signaling in different cellular systems. In the context of Salmonella infection of human colonic epithelial cells, increased ERK phosphorylation, NF-κB signaling, and IL-6 production/secretion was observed in PARP inhibitor (PJ-34) treated cells already at early timepoints. In untreated cells, both phosphorylation of ERK and nuclear NF-κB were detectable only at later timepoints^159^, suggesting that ADP-ribosylation prevents ERK signaling, downstream NF-κB activity, and IL-6 expression at an early timepoint of infection^159^.

In HeLa cells, extensive activation of PARylation by MNNG specifically decreased phosphorylation of ERK1/2, which ultimately induced cell death in an ERK-dependent manner^133^. A similar effect was observed in H_2_O_2_-induced ADP-ribosylation and apoptosis in cardiomyoblasts, which correlated with a reduction in both ERK and p38 phosphorylation^160^. PARP inhibitor administration (INH_2_BP) increased phosphorylation of ERK and p38 and cell viability, led to a downregulation of ROS and pro-apoptotic factors and increased levels of anti-apoptotic proteins^160^. Specific ERK and p38 inhibitors individually and in combination counteracted the pro-survival effect of INH_2_BP^160^, suggesting that the pro-survival effect of this PARP inhibitor could be ascribed to both MAP kinases. Of note, the ERK pathway had already been implicated in cardiac protection, while the pro-apoptotic or protective consequences of p38 signaling for the heart continue to be controversially discussed^161^. In addition, in cells and tissues other than heart an opposing effect of PARP inhibitor administration on the different MAP kinases can be observed. Inhibition of PARP1 increased ERK activation and counteracted cell death in the context of H_2_O_2_-induced apoptosis of human WRL-68 cells, while simultaneously decreasing p-JNK and p-p38 (as discussed in the “Positive regulation of MAPK signaling by ARTD family members” section)^148^. Similarly, a protective role of ADP-ribosylation inhibition has also been described in a model of retinal degeneration, where phosphorylation of ERK was increased, while p-JNK and p-p38 were decreased (as discussed in the “Positive regulation of MAPK signaling by ARTD family members” section) upon PARP inhibitor administration (HO3089)^146^. The mechanism for simultaneous upregulation of ERK and downregulation of JNK and p38 was not discussed in detail in these models of H_2_O_2_-induced cell death and retinal degradation. However, one could speculate that the protective effect of PARP inhibitors arises from increased pro-survival ERK signaling and simultaneous dampening of stress-induced JNK and p38 signaling.

From this point of view, it seems controversial that in H_2_O_2_-treated PARP1 knockout MEFs survival correlated with enhanced JNK and p38 phosphorylation, while p-ERK decreased in absence of PARP1 (as discussed in the section “Positive regulation of MAPK signaling by ARTD family members”)^144^. However, it is worth mentioning that the inhibition of JNK and p38 did not affect cell survival of H_2_O_2_-treated PARP1 knockout MEFs^144^. In addition, cell survival of H_2_O_2_-treated WT cells could not be rescued by MEK inhibitors^144^, indicating that cell viability is not affected by JNK and p38 nor by ERK signaling, respectively.

In contrast, in Langendorff perfused rat hearts, PARP inhibitor (L-2286) administration resulted in an increased activation in all three MAP kinases and protected from myocardial injury^162^. Similarly, L-2286 was also found to be protective in isoproterenol-induced myocardial injury^162^. However, in this case, PARP inhibitor administration correlated with increased p-ERK and p-p38 but decreased p-JNK^162^. Intriguingly, AKT phosphorylation was found to be elevated in both cases of myocardial injury^162^, which further suggests that AKT signaling might be an upstream ERK activator. Unfortunately, despite the wealth of correlative data collected, for most of the above discussed findings, the molecular mechanism(s) remain elusive. While the protective effect of PARP inhibitors seems to be applicable to a variety of different biological systems, in most studies they have not been further investigated to determine whether these protective effects are dependent on the observed changes in MAPK activity. Therefore, it is possible that changes in MAP kinase phosphorylation are merely a secondary consequence and not the cause of the observed phenotype.

ADP-ribosylation mediated inhibition of JNK1 signaling in multiple myeloma and hepatocellular carcinoma induced a pro-survival effect^138,163^. PARP14 was found to interact with and probably ADP-ribosylate JNK1, thereby inhibiting its kinase activity and suppressing JNK1-dependent apoptosis^138^. Similarly, PARP14 depletion or inhibition by PJ-34 resulted in increased JNK1 activity and cell death^138^. Moreover, in hepatocellular carcinoma cells, PARP14 promoted aerobic glycolysis by inhibiting JNK1-dependent phosphorylation of PKM2^163^. Since the unphosphorylated form of PKM2 is a positive regulator of the transition from oxidative phosphorylation to glycolysis, PARP14 might be a crucial mediator of the Warburg effect in hepatocellular carcinoma^163^. Albeit not in the context of JNK, the importance of PARP14 as an anti-apoptotic factor and regulator of glycolysis has also been discussed under different conditions^164^. Interestingly, by promoting aerobic glycolysis, PARP14 also increases the levels of the reducing equivalent NADPH^163^ and shifts the cellular redox state. On the other hand, aerobic glycolysis in cancer cells generally increases the cytoplasmic NAD^+^ pool, a phenomenon that might also be found in hepatocellular carcinoma. However, due to an extremely low *K*_m_ NAD^+^ PARP14 activity is possible under normal conditions and does not depend on an increase in NAD^+^.

## Interplay between AKT signaling and ARTD family members

### Overview of the PI3K/AKT pathway

**Fig. 5:**
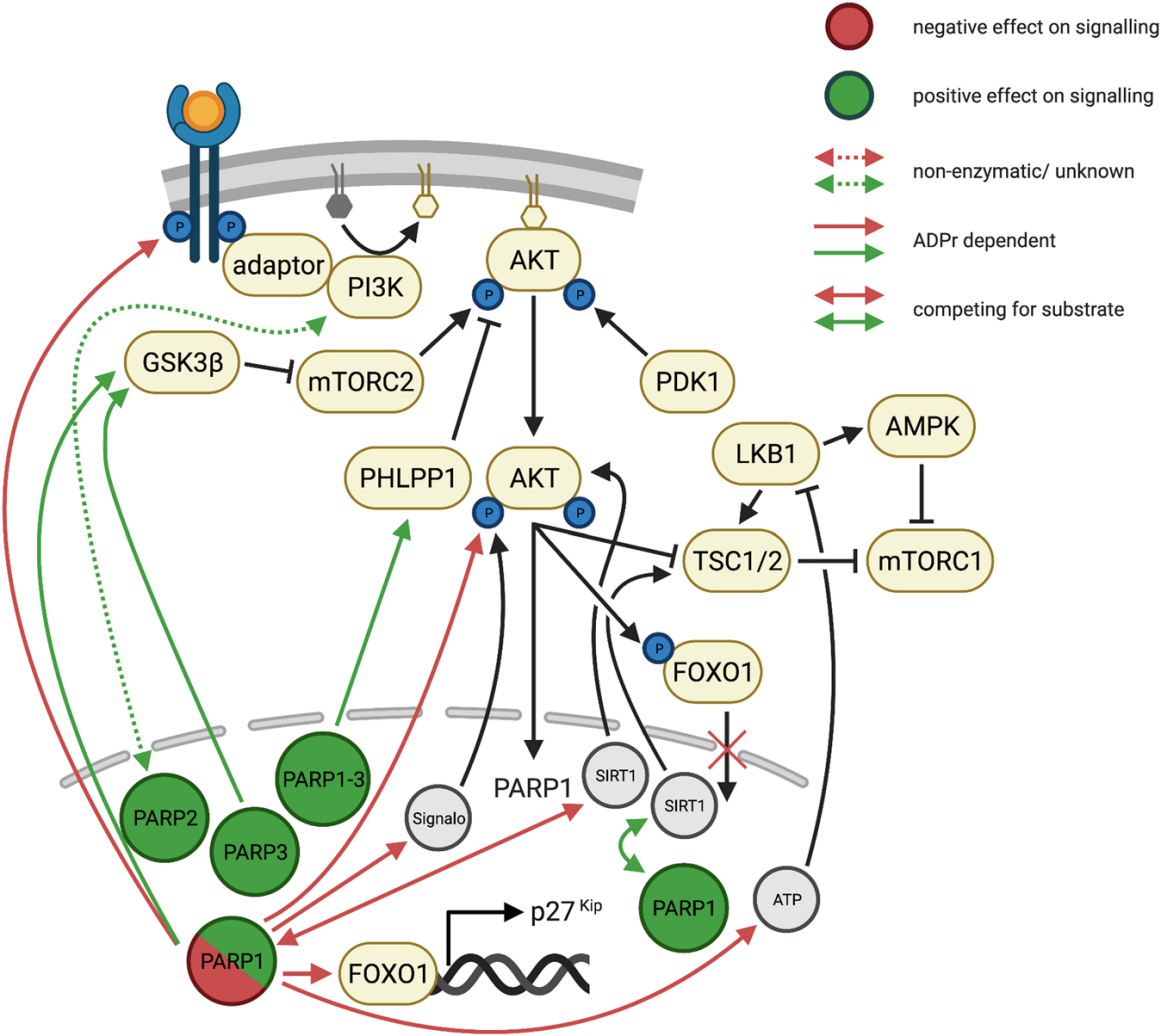
Schematic overview of the interplay between AKT signaling and ARTD family members. Positive regulations of AKT signaling by ARTD family members are depicted in green, while negative effects of ARTDs on the signaling pathway are shown in red. Solid lines indicate the contribution of ADP-ribosylation or competition for substrate in the regulation of AKT signaling. In case the contribution of ADP-ribosylation was not described or the protein itself rather than its enzymatic activity is involved in the regulation, the interactions are represented by dashed lines.

The serine-threonine kinase AKT (also called PKB) is a signaling molecule involved in regulating diverse biological processes including glucose metabolism, cell cycle, cell growth, and survival^165,166^. Moreover, activated AKT participates in control of altered glucose metabolism in cancer cells (i.e., aerobic glycolysis) and is sufficient to promote aerobic glycolysis^165^ which usually results in increased cytoplasmic NAD^+^ levels^117^. AKT is activated downstream of different receptor tyrosine kinases, cytokine receptors, and G protein-coupled receptors (Fig. 5)^165^. Receptor activation triggers the recruitment of phosphoinositide 3-kinase (PI3K), which binds with its SH2 domain to the phosphorylated tyrosine residues of the cytosolic receptor domains or to adaptor molecules^166,167^. This retains PI3K at the plasma membrane and, thus, allows phosphorylation of membrane-bound phosphoinositides^166,167^. AKT binds to the phosphorylated phosphoinositide and is phosphorylated by PDK1 at threonine residue T308^166,167^. AKT then becomes fully activated by mTORC2 via a second phosphorylation event at a distinct serine residue Ser473^166,167^. Fully activated AKT is involved in regulating diverse cellular processes by specifically phosphorylating multiple cytoplasmic substrates^166,167^. For instance, phosphorylation of the pro-apoptotic proteins BAD and FOXO counteracts apoptosis, while AKT-dependent phosphorylation of GSK3 changes the cellular metabolism by increasing glycogen synthesis^166^. Interestingly, AKT targets are implicated in the regulation of the MAPK pathway^166,167^ and AKT itself has been shown to suppresses the JNK and p38 pathway^166^. Moreover, AKT phosphorylates NAD kinase (NADK), which activates NADK and promotes the conversion of NAD^+^ to NADP^+^ and aerobic glycolysis^165^. Thus, AKT is directly involved in regulating the cellular metabolic homeostasis, modulating the availability of free NAD^+^ levels which in turn might reduce ADP-ribosylation activity.

### Influence of AKT signaling on ARTD family member expression

Only a few and opposing results are known about the regulation of ARTD family members by AKT signaling. While seizure induction increased PARP1 abundance and enzymatic activity in a certain type of astrocyte, in another type of astrocyte seizure led to a decrease in PARP1 and its enzymatic activity^131^. Inhibition of AKT signaling dampened PARP1 protein abundance and activity in both cases^131^. AKT might, thus, be involved in enhancing PARP1 expression and signaling.

### Positive regulation of AKT signaling by ARTD family members

PARP2 (ARTD2) expression in synovial cells from a rat rheumatoid arthritis model was found to be increased compared to control cells^168^. The increase in PARP2 led to enhanced cell proliferation, as well as increased phosphorylation of components of the AKT signaling cascade, including PI3K, AKT, and mTOR^168^, suggesting that PARP2 mediates cell survival and proliferation via AKT signaling. Interestingly, in HeLa cells, knockdown of PARP2 led to a decrease in total protein levels of PI3K, AKT, and mTOR, however the phosphorylation status was not analyzed^169^. Of note, tissue biopsies of cervical cancer patients displayed increased expression of PARP2 and components of the AKT cascade compared to non-tumor tissue^169^, illustrating the potential importance of this signaling network in vivo. Whether the observed effects depend on the ADP-ribosylation capacity of PARP2 or simply rely on the protein itself was not further elucidated. However, due to the comparatively high *K*_m_ NAD^+^ value of PARP2, it is more likely that it is the protein itself and not its enzymatic activity that contributes to the observed phenotype. Nevertheless, PARP2 ADP-ribosylation activity contributions cannot be completely excluded.

In BRCA1-deficient triple-negative breast cancer cell lines, the enzymatic activity of PARP3 (ARTD3) was involved in promoting cell survival and proliferation and correlated with increased AKT phosphorylation^170^. It was suggested that the activity of GSK3β, a negative regulator of the mTORC2 component *RICTOR*, was inhibited by PARP3-dependent ADP-ribosylation^170^. This resulted in enhanced expression of *RICTOR* and ultimately in increased phosphorylation of AKT by mTORC2^170^.

Inhibition of ADP-ribosylation by PARP inhibitors such as Olaparib, Rucaparib, and Niraparib was originally approved by the FDA for the treatment of BRCA-deficient tumors^171^. However, more recent evidence suggested that PARP inhibitor treatment could be applicable to a wide range of malignancies, including tumors with high replication stress and homologous recombination deficient cancers^172,173^. Prolonged inhibition of ADP-ribosylation in different human cancer cells either decreased phosphorylated AKT only^174,175^ or reduced both total and phosphorylated AKT protein^141^, which in turn reduced proliferation or induced cell cycle arrest. Although it was assumed that the observed cytotoxic effect was independent of the DNA repair capacity of the cell^174^, others emphasized increase in DNA damage as one of the primary causes of cell death^175^. Phosphorylation of FOXO3A by AKT, was reduced upon PARP inhibitor administration, allowing nuclear translocation and transcriptional activation of pro-apoptotic target genes^174^. Mechanistically, PARP inhibitors enhanced the phosphatase activity of the negative AKT regulator PHLPP1, which is responsible for the reduction in AKT Ser473 phosphorylation and subsequent inactivation^174^. Overall, these findings suggest that ADP-ribosylation-dependent enhancement of AKT signaling benefits cell survival and proliferation, thus confirming that PARP inhibitor administration in tumors might be beneficial even in BRCA-proficient tumors. Of note, there is yet another mechanism that adds complexity to the treatment with PARP inhibitors: independently of its effect on the cellular DNA repair capacity, PARP inhibitors have also been shown to promote cell surface PD-L1 levels^91,176,177^. Besides STAT3 (discussed in the “Negative regulation of JAK/STAT signaling by ARTD family members” section), GSK3β was proposed to be involved in regulating cell surface PD-L1 levels downstream of PARP1^176^. These data suggest that a combined treatment of PARP inhibitor and PD-1/PD-L1 blockade might be beneficial in cancer patients. Moreover, normalization of characteristics such as heart size and cardiac function in the hearts of diabetic rats was observed after inhibition of ADP-ribosylation by 1,25(OH)_2_D_3_^178^. Interestingly, in diabetic rats, PARP1 inhibition not only dampened PARP1 expression but also mTOR phosphorylation, which correlated with increased phosphorylation of TSC2 (a negative regulator of mTORC1), and enhanced expression of SIRT1^178^. Therefore a regulatory circuit was proposed that involves the positive regulation of TSC2 by SIRT1, which in turn is inhibited by PARP1 (potentially through depletion of the shared co-factor NAD^+^)^178^. Since prolonged mTOR activation can be detrimental^179^, it is possible that in the context of diabetic cardiomyopathy, inhibition of mTOR can confer a protective effect. Of note, the PARP inhibitor 1,25(OH)_2_D_3_ is an active form of vitamin D^180^ and diabetic patients often display vitamin D insufficiency^178^.

### Negative regulation of AKT signaling by ARTD family members

In contrast to the evidence presented above, PARP1-dependent ADP-ribosylation was also reported to inhibit the AKT signaling cascade and PARP inhibitor administration exerted a protective role in this context^146,151,162,178,181–187^. In cardiomyocytes, high glucose treatment-induced oxidative stress, and DNA damage increased the expression and activity of PARP1^183^. Silencing of PARP1 reduced the observed inflammatory response induced by high glucose treatment and protected cardiomyocytes against high glucose-induced apoptosis by activating the pro-survival AKT pathway through insulin-like growth factor 1 receptor^183^. In a diabetic cardiomyopathy mouse model, PARP1 knockout improved cardiac function as well as diabetic cardiomyopathy-induced structural changes^183^. In line with the high glucose treated cardiomyocytes in vitro, PARP1 deletion in mice reduced the concentration of proinflammatory cytokines, decreased apoptosis, and enhanced activation of IGF-1R and AKT in the diabetic cardiomyopathy model^183^. Similarly, in pathologies involving heart cells (different perfusion models in rat hearts), the cytoprotective effect of different PARP inhibitor treatments correlated with a significant increase in AKT phosphorylation^143,162^, suggesting that under these conditions ADP-ribosylation negatively regulates the PI3K/AKT signaling pathway. Of note, in models of heart pathologies, changes in MAP kinases were evident upon PARP inhibitor administration. However, there is no consensus on whether MAP kinases were decreased^143^ or increased^162^. Knockdown of PARP1 in hypoxia-stimulated rat cardiomyocytes and rat myoblasts resulted in decreased apoptosis and increased viability and phosphorylation of AKT^187^. The protective effect of PARP inhibitors also holds true for seizure in the hippocampus of rats that led to a considerable amount of ADP-ribosylation^186^. Inhibition of ADP-ribosylation by 3-aminobenzamide (3-AB) attenuated neuronal apoptosis in this model of epilepsy and correlated with increased activation of the pro-survival AKT signaling^186^.

In ocular pathologies, inhibition of ADP-ribosylation upon hypoxia/reoxygenation of rat eye or hypoperfusion-induced retinal degradation not only increased phosphorylation of AKT but also resulted in phosphorylation of downstream targets such as GSK3β. Furthermore, reversal of the induced histological changes was observed in both ocular pathologies^146,151^. Of note, in both pathologies the observed increase in AKT phosphorylation correlated with a decrease in JNK and p38 phosphorylation^146,151^. Furthermore, PARP inhibitor (3-AB or DPQ and PJ-34) administration after NMDA-elicited cell death in primary hippocampal neurons, and in a model for bladder dysfunction, counteracted cell death and correlated with enhanced AKT phosphorylation^181,182^.

Increased AKT phosphorylation and higher cell viability were also observed upon inhibition or silencing of H_2_O_2_-induced PARP1 in WRL-69 human liver cells^184,185^. An explanation as to how nuclear PARP1 could negatively influence the cytoplasmic AKT has been proposed: PARP1-dependent ADP-ribosylation counteracts the formation of a signalosome in the nucleus^185^. In contrast, inhibition of PARP1 activity allowed the formation of the signalosome, translocation to the cytoplasm, association with additional factors, and ultimately AKT phosphorylation and activation^185^. Moreover, PARP1 bound to and ADP-ribosylated FOXO1, thereby inhibiting FOXO1-induced transcription of p27^Kip^ ^188^. The latter examples highlight the fact that PARP1 influences PI3K/AKT signaling not just at one point, but at various levels throughout the cascade.

Inhibition of ADP-ribosylation or knockdown of PARP1 in lung cancer cell lines increased PI3K/AKT signaling, as well as global ATP levels^189^. In addition, PARP inhibitor (Olaparib, Rucaparib) administration decreased activity and expression of the stress sensor LKB1, as well as the phosphorylation states of LKB1 downstream targets AMPK and TSC2^189^. As both AMPK and TSC2 are negative regulators of mTORC1, this suggests that PARP inhibition drives PI3K/AKT signaling at least partly by increasing ATP availability which reduces stress response signaling via LKB1^189^. Interestingly, the sensitivity of different small cell lung cancer (SCLC) cell lines to the ADP-ribosylation inhibitor BMN 673 negatively correlated with the activity of the pro-survival pathway AKT. This suggests that cells with higher AKT activation were less sensitive to PARP inhibition^190^ and the increase of AKT pathway signaling could potentially depict an attempt of SCLC cells to escape PARP inhibition^189^. Similarly, the combination of a cytotoxic drug and inhibition or knockdown of PARP1 rendered human bladder carcinoma cells more resistant to apoptosis compared to treatment with a cytotoxic drug only^191^. Moreover, inhibition or knockdown of PARP1 correlated with increased phosphorylation of AKT and AKT’s downstream target GSK^191^. Of note, inhibition of the AKT pathway reversed the beneficial cytoprotective effects observed in PARP inhibitor-treated cells, confirming that PARP inhibition-induced AKT pathway activation could be responsible for the increased viability observed in these cancer cells^191^. In this case, a combination of both PARP inhibitors and PI3K/AKT pathway inhibitors might be beneficial in anti-tumor therapy^189,191^. Based on the data discussed above, it is evident that AKT activity correlates with survival. Controversially, the opposite was found in malignant pleural mesothelioma^192^. Inhibition of ADP-ribosylation decreased cell viability while simultaneously correlating with increased phosphorylation of pro-survival protein AKT^192^. The phosphorylation of a downstream effector of AKT, mTOR, was decreased^192^. It was speculated that inhibition of ADP-ribosylation increased the nuclear NAD^+^ concentration, allowing SIRT1 to de-acetylate AKT, which is then phosphorylated^192^. However, a second role of SIRT1 was discussed; by interacting with TSC1/2 (negative regulator of mTORC1) the phosphorylation of mTOR was inhibited^192^. This dual role of SIRT1 could explain why high AKT phosphorylation cannot necessarily be translated into mTOR activity.

## Concluding remarks and perspectives

The importance of signaling events as a hallmark for a plethora of cellular processes in health and disease has become apparent in the last decades. However, the influence of intracellular ARTD family members mediated ADP-ribosylation on signaling has only recently emerged. Importantly, beyond the regulation of signaling pathways by ADP-ribosylation, the non-enzymatic properties of ARTD family members can also impact signaling as described in multiple examples throughout this review. So far, PARP1 is the most studied member of the ARTD family, due to its abundance and involvement in genomic integrity. Nonetheless, a growing body of evidence is supporting the importance of the other ARTDs for intracellular signaling. As discussed here, the effect of a single ARTD member on a signaling cascade can be both inhibitory and/or activating. Despite the abundance of studies showing a correlation between ARTD family members and signaling events, the precise mechanism of regulation as well as the contribution of the enzymatic activity often remains elusive or is under debate. Acquiring more detailed insights into the context-specific mechanism of action will help us to gain an understanding of this post-translational modification in intracellular signaling. While many studies focus on elucidating the impact of ARTDs on cellular function, little is known about the transcriptional regulation of ARTD family member. Additional efforts are thus required to understand these cross-talks and regulations. One prominent exception for this aspect are the ARTD family members induced in response to a viral infection^7,73^. Their induction suggests that they play a role in the antiviral defense. Indeed, several ARTD family members are found to restrict viral replication^73^. On the other hand, viruses encode both ARTs as well as ADP-ribosylhydrolases^193^. For example, viral macrodomains have been described to interfere with the innate immune response^193^. This provides evidence for an ancient and central role of ADP-ribosylation in host-pathogen interactions and suggests a co-evolution of ARTs.

Furthermore, ADP-ribosylation and ARTDs can promote immune signaling as well as the inflammatory response and subsequently promote or suppress chronic inflammatory diseases and oncogenic phenotypes. Besides the well-established use of PARP inhibitors in anti-tumor therapy, beneficial effects of PARP inhibitor administration were demonstrated in various inflammatory disease models including myocardial infarction and different auto-immune diseases^85^.

NAD^+^-bioavailability is the most basic requirement for the regulation of signaling cascades by ADP-ribosylation. Therefore, synthesis as well as spatial compartmentalization and temporal changes in cellular NAD^+^ concentration might strongly influence the enzymatic activity of ARTD family members and subsequently their signaling output. Since the affinities of the known ARTD family members for NAD^+^ are quite different (Table 1), local NAD^+^ changes (e.g., by association with enzymes synthesizing NAD^+^ ^194–196^ or stress-induced redistribution of NAD^+^ itself^197^), very likely contribute to their activity. In addition, co-factors might influence NAD^+^ and substrate affinity and, thus, alter the ADP-ribosylation profiles of a particular cell^198^. Since the unbound NAD^+^ exceeds free NADH by 600–1000 times in the cytoplasm but only 7–8 times in the mitochondria, metabolic changes affecting NAD^+^/NADH ratio might have a much bigger effect on NAD^+^ availability in the mitochondria. This suggests that the recently described mitochondrial ADP-ribosylation^197^ is likely more tightly coupled to metabolic changes (e.g., switch from oxidative phosphorylation to aerobic glycolysis) compared to nuclear and cytosolic ADP-ribosylation. The roles of various ARTs are not only dependent on their catalytic activities, but also on non-catalytic activities (e.g., complex formations). Whether the latter ultimately impacts NAD^+^-bioavailability is currently not clear. The generation of biosensors capable of measuring free NAD^+^ in cells has enabled studies examining compartmentalized NAD^+^ concentration in all cellular compartments. In future studies, it will be interesting to determine cellular NAD^+^ levels under specific physiological and pathophysiological conditions to understand whether NAD^+^ availability does indeed provide a framework for enzymatic activity of ARTD members.
